# Efficacy of the NaviTip FX irrigation needle in removing calcium hydroxide from root canal

**DOI:** 10.4317/jced.50857

**Published:** 2012-10-01

**Authors:** Clovis M. Bramante, Bethânia C. Pinheiro, Roberto B. Garcia, Alexandre S. Bramante, Norberti Bernardineli, Ivaldo G. de Moraes, Marco A. Húngaro-Duarte, Tiago N. Pinheiro

**Affiliations:** 1 Professor of Endodontics, Bauru Dental School, University of São Paulo, USP, Bauru- SP, Brazil.; 2 Professor of Dentistry, UNIP Dental School, Manaus-AM, Brazil.; 3 Professor of Oral Pathology and Oral Medicine, Amazonas State University - Dental School, Manaus-AM, Brazil.

## Abstract

Objective: To evaluate the effectiveness of the NaviTip FX, brush-covered irrigation needle, in removing calcium hydroxide from the root canal. Study Design: Thirty single-rooted teeth were randomly divided into three groups: A - irrigation with a hypodermic needle inserted as far as possible without binding and activation with #30 K-type file; B - Irrigation with a hypodermic needle without activation; C - irrigation with NaviTip FX needle. Sodium hypoclorite 1% was used in irrigation. The root canals were examined trough scanning electron microscopy. Calcium hydroxide removal was recorded at 1, 5, and 10mm from the working length (WL) and the data were analysed using one-way ANOVA test (p<0.05). Results: NaviTip FX and hypodermic needle activated with #30K-type file showed lower score at 10 and 5mm with no significant difference between them. Comparison within groups did not show significant differences. All groups showed significantly better smear layer removal at 5 and 10 mm from the WL. Conclusion: The apical third (1mm) of the root canal was found to be the most critical site for Ca(OH)2 removal.

** Key words:**Calcium hydroxide, irrigation, scanning electron microscope, NaviTip FX.

## Introduction

Calcium hydroxide has been shown to be an effective intracanal medicament during endodontic therapy. Its effects are seen through antimicrobial action, the inhibition of osteoclastic activity and a favorable tissue repair response (1-4). The removal of calcium hydroxide is usually accomplished through several irrigating rinses in conjunction with hand instrumentation. Saline, sodium hypochlorite, EDTA and combinations have been used as irrigating with varying results. Other studies have found that calcium hydroxide removal was difficult or incompletely removed (5-8).

Residual Ca(OH)2 is potentially affected by remnants of smear layer and pulpal debris remaining from instrumentation. Curvature of the canal, type of irrigating used, and depth of penetration of that irrigating will also affect Ca(OH)2 removal (9,10). Ultrasonic instrumentation of the canal has been widely advocated as an effective modality for cleaning pulpal remaining and dentinal debris from canals and isthmus. The mechanical agitation provided by ultrasonic instrumentation or a rotary file in conjunction with irrigation may also enhance removal of Ca(OH)2 (10).

To aid in root canal debris removal, a few attempts have been described that use cotton-wrapped around an endodontic file or a broach (11), or the use of an Endobrush (12). The former study indicated that a cotton-wrapped around a file or broach was not able to clean the canal properly especially the irregularities, whereas, the later study demonstrated a better cleaning effect when the Endobrush was used with hand instrumentation compared with that of instrumentation alone.

Recently, an irrigation needle covered with a brush (NaviTip FX, Ultradent, UT) was introduced into the mar-ket.The literature have reported the use of needle NaviTip FX in removing smear layer (13-16). However, never been reported in the removal of calcium hydroxide from root canal. Therefore, the objective of this study was to evaluate the effectiveness of NaviTip FX in removing calcium hydroxide from root canal using scanning electron microscopy.

## Material and Methods

Thirty noncarious extracted human premolars with single root canals, fully developed apices, and straight roots were included in this study. The teeth were radiographed to confirm canal patency and complete root formation. Standard endodontic access cavity preparations were performed to the pulp chambers. After access a #15 k-type file was inserted into the canal until the tip was just visible at the apical foramen. The length of the file was measured and 1mm was subtracted from this length to establish working length (WL). Preliminary coronal enlargement was achieved using Gates-Glidden drills of sizes #3, 2, and 1. The canals were instrumented with 0.04 taper ProTaper nickel titanium rotary files (Dentsply Maillefer, Ballaigues, Switzerland) using a crown-down technique to a size #40 master apical file.

Calcium hydroxide paste with saline solution as vehicle was inserted in the root canals using a Lentulo spiral throughout the working length. Gutta- percha and IRM was used to close de access cavity and the teeth remained inside a humid chamber at 36ºC for 7 days.

The teeth were then randomly divided into three groups of 10 teeth each according to the irrigation protocol: group A- irrigation with a #30-gauge hypodermic needle inserted as far as possible without binding and activation with #30 K-type file; group B-irrigation with a #30-gauge hypodermic needle without activation; group C-irrigation with #30-gauge NaviTip FX (Ultradent Products Inc, South Jordan, UT). Sodium hypoclorite 1% was used to irrigate the canals after each instrument use.

The teeth were grooved vertically with a carborundum disc on the buccal and lingual surfaces. The teeth were then divided them into two halves with a chisel and mallet. The half with most visible part of the apex was used for scanning electron microscopic (SEM) evaluation. Each sample was divided into three equal parts (apical, middle, and coronal). The samples were dried, sputter-coated with gold using fine-coat ion sputter BAL–TECH SCD 050 (Fine coat ion sputter, BAL–TECH) and then evaluated using the SEM (JEOL JSM-T 220A, JEOL Ltd.) with magnification 200x. Calcium hydroxide on the canal wall was evaluated using the following sco-ring:1-clean root canal, only few small particles; 2-few small isles of CaOH covering less than 25% of the root canal wall; 3-many accumulations of CaOH covering more than 25% but less than 50% of the root canal wall; 4-more than 50% of the root canal wall covered by CaOH. The results were statistically analyzed using the ANOVA at significance level p<0.05.

## Results

Mean amounts of debris recorded at the apical, middle, and coronal in the 3 experimental groups are listed in  Table 1. Of a possible maximum of 5, mean debris scores in all 3 groups ranged from 4.6±0.699 to 2.9±0.994 ( Table 1). The canals irrigated using the NaviTip FX needle showed lower average scores at 10 and 5mm, except at 1mm (apical third), were it showed the worst average compared to other groups.

**Table 1:**
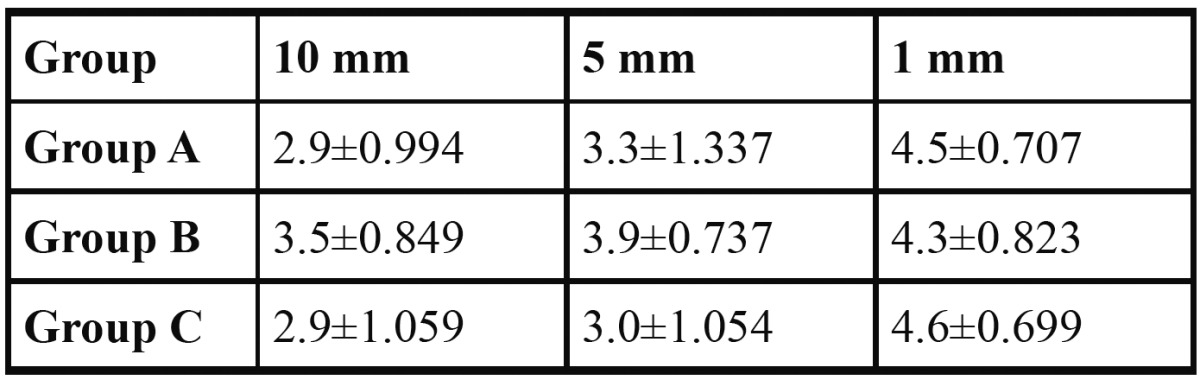
Mean debris scores (±SD) for the different groups at various levels (mm).

One-way ANOVA test depicted significant difference between the different levels on each group compared separately ( Table 2). When each level were compared among the different groups and when all levels were compared together between the studied groups there were no significant statistical difference on smear layer scores.

**Table 2:**
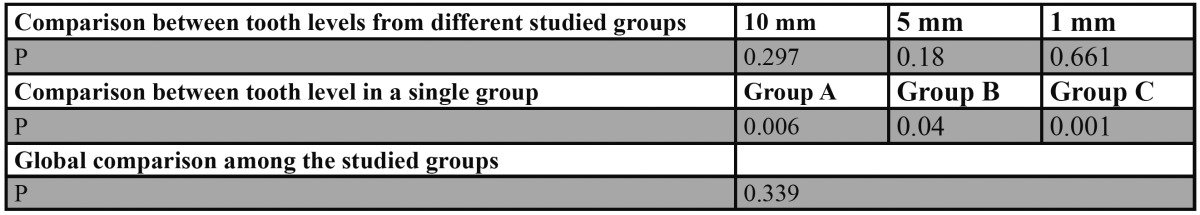
One-way ANOVA results for the following comparisons: Between levels from different groups, between levels in a single group and global comparison among the studied groups.

## Discussion

Chemomechanical instrumentation, or the mechanical and chemical cleansing of the root canal system, is a fundamental principle of root canal therapy. Calcium hydroxide is the most used medication in endodontic treatment because antibacterial activity (1-4). Intracanal Ca(OH)2 is usually removed from the root canal by the use of copious irrigation with sodium hypoclorite or saline combined with instrumentation and final rinse with 17% EDTA. However, none of the techniques mentioned above were efficient in removing all the material from root canal. The residual calcium hydroxide may interact with the root canal sealer and interfere with its sealing ability (17-22). The aim of this study was evaluate different methods for calcium hydroxide removal using 1% sodium hypoclorite as irrigant solution. Three experimental groups were proposed, a #30-gauge hypodermic needle with and without activation using a #30 K-file, and a #30-gauge NaviTip FX needle. The only significant statistical difference observed was related to the differences among radicular level within each group. Our expectation was that the use of NaviTip FX our activation with a #30 K-type file could improve the debris into suspension removal. However, our findings do not support this hypothesis.

All of the irrigation methods successfully flushed loose superficial debris of calcium hydroxide from the root canals. Only small amounts of superficial debris could be found on the canal walls of the specimens. It is known that residual calcium hydroxide influences the setting mechanism of zinc oxide-eugenol-type endodontic sealers (20). The short-term clinical implications were a rapid setting reaction of the sealer that blocked gutta-percha entrance and placement to full working length. The NaviTip FX needle showed impressive results regarding smear layer and debris removal after root canal instrumentation, verified by various studies (13-16). However its ability in remove calcium hydroxide debris, did not reached such results.

The present study used 1% sodium hypochlorite as the only irrigating solution. It is well known the benefits of a additional EDTA flush in the removal of inorganic mater present on the smear layer. Further studies should test an extra irrigation phase using EDTA on each proposed irrigation methods in order to evaluate its additional influence on calcium hydroxide debris removal. If this kind of experiment proves that EDTA irrigation is significantly efficient, it would prove that the irrigation solution regimen is just as important as the irrigating instruments applied in the operation.

## Conclusion

None of irrigation protocol were able to completely remove the calcium hydroxide. NaviTip needle remove Ca(OH)2 similarly to hypodermic needle with or without activation with K type file. The apical third of the root canal was found to be the most critical site for Ca(OH)2 removal.
